# L'intervention de Castaing dans le traitement chirurgical de l'instabilité chronique latérale de la cheville (à propos d'une série continue de 52 cas)

**DOI:** 10.11604/pamj.2014.18.288.3772

**Published:** 2014-08-12

**Authors:** Jalal Boukhris, Rifi Mojib, Sami Mezghani, Jean Henri Jaeger

**Affiliations:** 1 Service de Chirurgie Orthopédique du Genou et Traumatologie du Sport- CHU Strasbourg, Strasbourg, France

**Keywords:** Cheville, instabilité chronique, Castaing, ankle, chronic instability, Castaing

## Abstract

L'instabilité chronique de la cheville est l'une des évolutions possibles dans l'histoire naturelle de l'entorse aigüe de cheville. Elle représente 10% à 30% des séquelles d'entorses. Le traitement chirurgical est réservé aux patients ayant une instabilité de cheville persistante après rééducation à l'origine d'une gêne invalidante. La technique de ligamentoplastie utilisant le court fibulaire selon Castaing est la plus répondue en France. La simplicité du geste et ses bons résultats nous ont incités à poursuivre cette voie. Il s'agit d´une étude rétrospective à propos de 52 patients ayant bénéficié d'une ligamentoplastie au court fibulaire selon Castaing entre Janvier 2000 et Décembre 2010. Le recul minimum était d'un an. L’évaluation des patients reposait sur le score de Karlson et le “Ankle score” Molander et Olerud. Les patients sont satisfaits ou très satisfaits de l'intervention dans 80% des cas revus. Les résultats fonctionnels de nos patients sont comparables à ceux publiés dans la littérature. Le prélèvement du transplant du court fibulaire ne semble pas avoir de retentissement sur la stabilité de la cheville. Les patients conservent une éversion du pied satisfaisante avec une force comparable au côté opposé. L’évolution arthrosique de la cheville ne semble pas significative après cette chirurgie. Le point faible de notre étude est le nombre des patients perdus de vue, cela est lié à la jeunesse de la population opérée et au recrutement du service d'envergure nationale.

## Introduction

L'instabilité chronique de la cheville est l'une des évolutions possibles dans l'histoire naturelle de l'entorse aigue de cheville. Elle représente 10% à 30% des séquelles d'entorses. Le traitement chirurgical est réservé aux patients ayant une instabilité de cheville persistante après rééducation à l'origine d'une gêne invalidante. La technique de ligamentoplastie utilisant le court fibulaire selon Castaing est la plus répondue en France. La simplicité du geste et ses bons résultats nous ont incités à poursuivre cette voie.

## Méthodes

**Figure 1:**
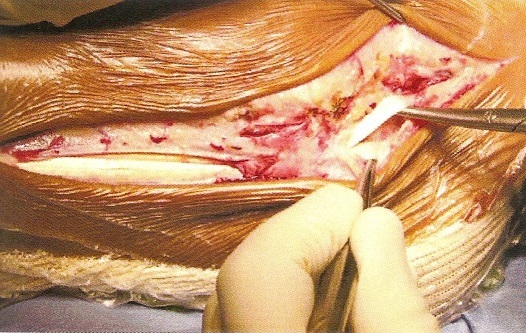
Incision cutanée et repérage du court fibulaire

**Figure 2:**
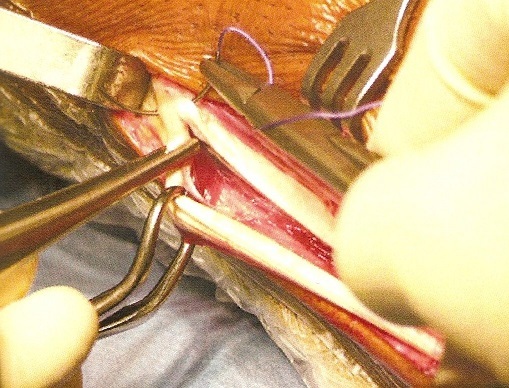
Suture proximale du tendon court fibulaire

**Figure 3:**
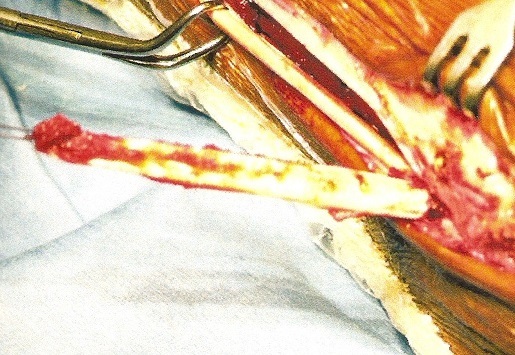
Transplant du court fibulaire

**Figure 4:**
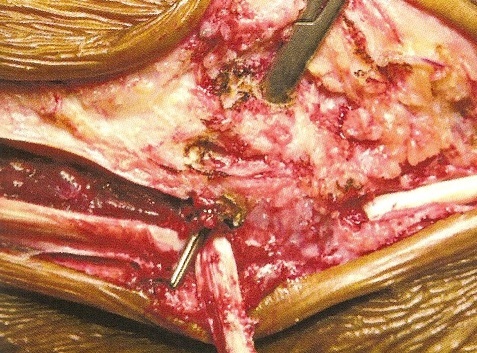
Tunnel malléolaire lateral

**Figure 5:**
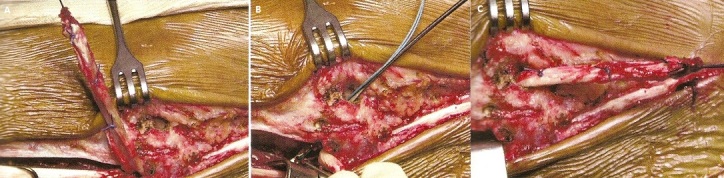
A, B, C: Progression du transplant dans le tunnel malléolaire

**Figure 6:**
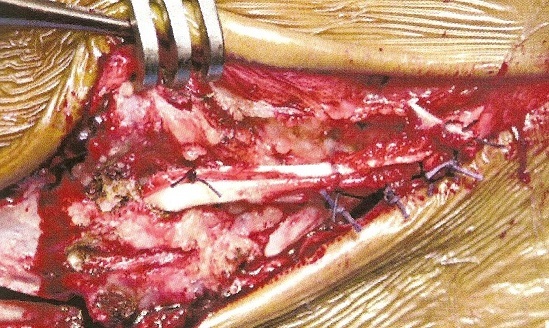
Suture du transplant sur lui-même

**Figure 7:**
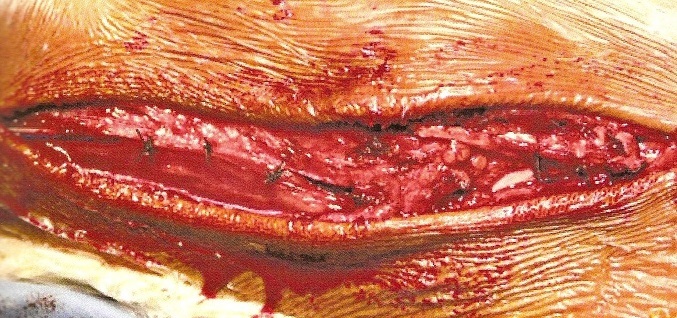
Fermeture de la gaine des fibulaires

**Description de la technique :** L'incision cutanée se fait sur le bord externe de la cheville (Figure 1). On repère le transplant du court fibulaire (Figure 2) et on le suture en proximal au tendon long péronier (Figure 3). Après réalisation du tunnel malléolaire latéral (Figure 4), on fait progresser le transplant à travers ce tunnel (Figure 5), puis on le suture sur lui-même avec une légère tension afin de corriger la laxité latérale de la cheville (Figure 6). On termine en suturant la gaine des fibulaires (Figure 7), sous peau et le plan cutané. La cheville est immobilisée dans une attelle provisoire.

**Description de la série :** Il s'agit d´une étude rétrospective à propos de 52 patients ayant bénéficié d'une ligamentoplastie au court fibulaire selon Castaing entre Janvier 2000 et Décembre 2010. Le recul minimum était d'un an. L’évaluation des patients reposait sur le score de Karlson, le “Ankle score” Molander et Olerud, la reprise des activités professionnelles et sportives ainsi que la satisfaction par rapport à l'intervention. Elle reposait également sur les clichés radiologiques standards de la cheville et des clichés en autovarus bilatéraux et comparatifs.

## Résultats

Nous avons revu 40 patients sur les 52 opérés avec un recul moyen de 60 mois. Nous déplorions 12 perdus de vue (patients injoignables ou ayant déménagés). La reprise du travail s'est faite avec un délai moyen de 9,1 semaines en moyenne (mini 4, maxi 20, E-T 5,9). Sur les 30 patients pratiquant un sport de loisir ou en compétition 26 patients (86% des cas) ont repris le sport qu'ils pratiquaient avant l'intervention mais seulement 21 d'entre eux ont repris au même niveau (80% des cas). Un patient a arrêté à cause d'une instabilité résiduelle, les 3 autres pour des raisons personnelles autres que la cheville. Les résultats subjectifs montraient 84% de patients satisfaits et très satisfaits et 2 patients déçus de l'intervention du fait de douleurs résiduelles.

Le score de Karlson moyen était de 84% avec les résultats suivants: 27 patients ayant un score bon ou excellent (score ≥ 80); 9 patients ayant un score moyen (60≤ score < 80); 4 patients avec un mauvais résultat (score < 60)

Le score fonctionnel de Molander et Olerud moyen était de 83,7% avec la répartition suivante: 26 patients ayant un score bon ou excellent (score ≥ 80); 9 patients ayant un score moyen (60≤ score < 80); 5 patients avec un mauvais résultat (score < 60)

Les patients ont estimé que leur cheville valait en moyenne 85,8% (mini 50%, maxi 100%) d'une cheville saine. Du côté opéré l'angle tibio-talien moyen était de 3,2° (mini 0°, maxi 8,8° E-T 2,15) pour un angle de 2,2° (mini 0°, maxi 5° E-T 1,74) en controlatéral. Nous avons constaté un cas d’évolution arthrosique sur la partie latérale de l'articulation tibio-talienne à type de pincement localisé et de condensation sous-chondrale. Cela concernait le patient le plus âgé de la série. Aucun patient ne présentait de signes d'arthrose de l'articulation sous-talienne sur les clichés radiologiques de profil.

## Discussion

Nous avons choisi la technique de Castaing car elle était simple et reproductible. Elle donne des résultats fonctionnels satisfaisants comme le montre notre travail et l’étude de la littérature. Le score de Karlsson moyen était de 84 et le score fonctionnel de Molander et Orlerud moyen de 83,7 points. Dubranne [1] dans sa série trouvait un score fonctionnel de Molander et Orlerud moyen de 85,5 points ce qui est comparable à notre étude.

Nous avons analysé les différents signes fonctionnels des patients tels la douleur, la stabilité, la reprise de la pratique sportive. Ces résultats étaient comparés aux séries de Castaing ou d'hémi-Castaing publiées dans la littérature.

Au dernier recul, la douleur était présente dans 26,9% des cas. 71% des patients algiques signalaient des douleurs peu importantes durant ou après la pratique sportive. Les séries anciennes ou récents de la littérature [1–3] trouvaient la présence de douleurs dans 37 à 58,7% des cas survenant comme dans notre série majoritairement à la pratique sportive.

Dans la littérature les séries de plastie au court fibulaire selon Castaing ont rapporté une bonne stabilité de la cheville, avec une instabilité persistante chez seulement 9 à 20% des patients [1, 3, 4]. Avec un taux d'instabilité résiduelle de 23% nous étions un peu au dessus des taux relevés dans la littérature. Cela pouvait être dû au fait que nous avions réalisé des plasties serrées pour ne pas enraidir l'articulation sous-talienne selon les enseignemet de Pascoët [5].

Le taux de patients reprenant le sport au même niveau était équivalent avec les techniques anatomiques [1, 6–9]. L'enraidissement de l'articulation sous-talienne, imputé aux ligamentoplastie, n'apparaissait pas comme un facteur limitant la reprise de la pratique sportive. En effet, la reprise sportive de notre série est comparable à celle de toutes les techniques de stabilisation de la cheville qu'elles soient anatomiques ou non.

Le résultat objectif de la technique était évalué par les clichés en autovarus. Nous avons observé une diminution de l'angle tibio-talien passant de 10,9° à 3,4°; soit une diminution de plus de 7° de l'angle tibio-talien. Cette réduction semblait plus importante dans les techniques de ligamentoplastie que dans les techniques de rétention capsulaire publiées [10]. Cela peut être en rapport avec le taux légèrement plus important d'instabilité résiduelle observé après réparation selon une technique anatomique [11].

L'analyse radiologique après ligamentoplastie de notre série avait mis en évidence un cas d'arthrose tibio-talienne latérale chez le doyen de la série (69 ans) mais pas d’évolution arthrosique de l'articulation sous-talienne.

Le taux de satisfaction des patients est resté toujours important (84%) malgré les douleurs résiduelles. Les patients avaient une cheville stable et pouvaient reprendre nombre de leurs activités sans appréhension, ce qui était leur motif de consultation initiale. Dans notre série seulement 2 patients s'estimaient déçus de l'intervention car ils conservaient des douleurs latérales de la cheville, et ceci malgré une cheville objectivement stable.

Au vu de nos résultats cliniques et de la littérature le sacrifice du tendon du court fibulaire ne semble pas entrainer de morbidité et de perte de force d’éversion, 33 patients sur 40 avaient une éversion estimée à 5/5. Une étude isocinétique menée par Colombet et al. [12] après une plastie de Chrisman-Snook au court fibulaire n'a pas montré une différence significative avec la cheville non opérée.

La dégradation des résultats observée avec la technique d'Evans [13] ne semblait pas se produire avec la plastie de Castaing. Notre série avec un recul d'environ 6 ans a montré des résultats durables dans le temps, et au-delà de la période à risque décrite par Karlsson [13], qui a montré que la distension se faisait entre 2 et 6 ans.

## Conclusion

Au total, la plastie stabilisatrice de Castaing “desserée” au court fibulaire apparaît encore actuellement comme une intervention de choix dans le traitement des laxités externes de la cheville et de l'articulation sous talienne chez le non sportif comme chez le sportif. Le point faible de notre étude est le nombre des patients perdus de vue, cela est lié à la jeunesse de la population opérée et au recrutement du service d'envergure nationale.
